# Strategies of selective changing: Preparatory neural processes seem to be responsible for differences in complex inhibition

**DOI:** 10.1371/journal.pone.0214652

**Published:** 2019-04-18

**Authors:** Stephanie Antons, Maren Boecker, Siegfried Gauggel, Vera Michaela Gordi, Harshal Jayeshkumar Patel, Ferdinand Binkofski, Barbara Drueke

**Affiliations:** 1 Institute of Medical Psychology and Medical Sociology, University Hospital of RWTH Aachen University, Aachen, Germany; 2 Department of General Psychology: Cognition and Center for Behavioral Addiction Research, University of Duisburg-Essen, Duisburg, Germany; 3 Division for Clinical Cognitive Sciences, Department of Neurology, Medical Faculty, RWTH Aachen University, Aachen, Germany; Technische Universitat Dresden, GERMANY

## Abstract

Selective inhibition describes the stopping of an action while other actions are further executed. It can be differentiated between two strategies to stop selectively: the fast but global *stop all*, *then discriminate* strategy and the slower but more selective *first discriminate*, *then stop strategy*. It is assumed that the *first discriminate*, *then stop* strategy is especially used when information regarding which action might have to be stopped is already available beforehand. Moreover, it is supposed that both strategies differ in matters of basal ganglia pathways used for their execution. Aim of the present study was to investigate the use of the two strategies in situations requiring selective changing of an action. Eighteen healthy male participants performed a selective stop-change task with informative and uninformative cues during fMRI. Behavioral results show that informative cues led to a benefit in both inhibition times and selectivity. FMRI data revealed that the same cortico-subcortical pathway was used with informative and uninformative cues. Behavioral and neuronal results indicate that participants used the *first discriminate*, *then stop* strategy for selective inhibition irrespective of the amount of previously available information. Moreover, the neural activity data indicate that the benefit in the informed condition was produced by an efficient preparation for the concrete change process. Possible factors that might affect which strategy is used for selective stopping are the level of previously available information (foreknowledge) and the experimental set-up, as e.g. task complexity.

## Introduction

In our daily life we frequently have to adjust our behavior in view of changing environments. Many situations require the selective inhibition of one specific action while other actions have to be further executed (e.g. stop walking, but still talking). Selective inhibition can be achieved through two mechanically different strategies (Aron, 2011): (1) the global inhibition strategy which can be described as *stop all*, *then discriminate* and (2) the true selective *first discriminate*, *then stop* strategy. At a first sight, both strategies behaviorally lead to the same result: the stopping of an action while other actions are further executed. Differences between the strategies become apparent in the time needed to inhibit an action (inhibition time) and the interference of stopping or changing one action with the ongoing action (selectivity). The *stop all*, *then discriminate strategy* leads to a fast but global stopping process which interferes with the execution of all other ongoing actions. This strategy is comparable with a short freezing of all actions before the action, which has to be continued, is re-initiated. By contrast, the *first discriminate*, *then stop* strategy is slower in inhibiting an action but leads to less interference with other actions. On a neural level, both strategies are assumed to be realized by the same brain network consisting of the right inferior frontal gyrus (rIFG), presupplementary motor area (preSMA), basal ganglia (BG), thalamus, and primary motor cortex (M1) [1]. However, Aron [1] assumes that the differences in reaction times and selectivity between the two strategies result from different processing routes within the BG.

There are three well known BG pathways which send information from cortex regions to the thalamus which again engages an excitatory or inhibitory effect on cortex regions [1–3]: the direct, indirect, and hyperdirect pathways. The direct pathway is assumed to be the motor promoting pathway which acts to increase the effect of the thalamus on cortical regions via inhibitory projections from the putamen and internal globus pallidus. It is assumed that this inhibition of the thalamus via the direct pathway leads to the release of appropriate cortically mediated behavior. By contrast the indirect and hyperdirect pathways has more inhibitory effects on cortically mediated behavior [1]. The indirect pathway includes the caudate nucleus and external part of the globus pallidus. Both nuclei have inhibitory efferences which finally lead to less inhibition of the internal globus pallidus and therefore to an increased inhibitory effect on the thalamus. The hyperdirect pathway includes the subthalamic nucleus (STN) which has an excitatory effect on the internal globus pallidus and accordingly leads to an increased inhibition of the thalamus. The somatotopic organization of structures in the indirect pathway and the higher number of synapses allow the selective inhibition of a single action [3]. The STN, in contrast, results in the inhibition of large thalamic areas and is therefore less selective. However, the bypassing of the striatum in the hyperdirect pathway and the smaller number of synapses involved, may result in a faster processing compared to the indirect pathway. Thus, it is assumed that the hyperdirect pathway enables the *stop all*, *then discriminate* strategy and the *first discriminate*, *then stop* strategy is processed via the indirect pathway [1].

Besides these different neural processing routes, it is presumed that the two strategies are used in different situations [4–6]. The *stop all*, *then discriminate* strategy focusses on inhibition speed and might be used in situations in which speed is more important than selectivity or in situations in which a selective inhibition is not possible because of missing information about the concrete action which has to be inhibited [5]. Previously available information (foreknowledge) about the action which possibly has to be stopped is assumed to trigger the *first discriminate*, *then stop* strategy, while the *stop all*, *then discriminate* strategy is probably used for reactive selective inhibition in situations without concrete information [4].

An eligible experimental paradigm for examining strategies to stop selectively is the stop-signal task (SST)[7]. Selective inhibition can be investigated using variants of the standard SST which require a bimanual response in go trials (e.g. pressing two buttons simultaneously, each with one of the index fingers) and the inhibition of only one response option in the case of a stop trial (e.g. stop pressing the right button with the right index finger, but still press the left button with the left index finger). Moreover, informative (foreknowledge) and uninformative cues can be used to trigger the different strategies to stop selectively.

In a first behavioral study, Aron and Verbruggen [4] were able to show that foreknowledge in a bimanual stop-signal task leads to longer inhibition times but less interference of the ongoing action compared to trials without foreknowledge. Findings by Aron and Verbruggen (4) seem to indicate, that the *first discriminate*, *then stop* strategy is predominantly used in informed conditions and the faster but less selective *stop all*, *then discriminate* strategy is used in uninformed conditions. These results were replicated in a study by Claffey, Sheldon [5]. Additionally, the authors used transcranial magnetic stimulation to test whether foreknowledge activates a control set for the action which might need to be inhibited in the future. The measured motor evoked potentials indicated that participants used foreknowledge to apply advanced control (already during preparation phase) onto the specific motor representation of the response which had to be inhibited. These findings underpin the assumption by Aron and Verbruggen [4] that foreknowledge in a stop-signal task may lead to the use of the *first discriminate*, *then stop* strategy which presumably is processed via the indirect BG pathway and that participants use the *stop all*, *then discriminate* strategy and presumably the hyperdirect pathway in uninformed situations. However, to further support these assumptions, more studies, especially with neuroimaging techniques, are needed.

The aim of the following study was to investigate the neural correlates of the two mechanisms to stop selectively, namely the *stop all*, *then discriminate* and *first discriminate*, *then stop* strategies. Accordingly, we used a SST variant which included informative and uninformative cues to trigger the selective stopping strategies during fMRI. Moreover, we simulated action control situations which do not only require an inhibition of action but also a reengagement of another action. In previous studies [8–10] it has been shown that this stop-change task (SCT) contains three processes: the go-, stop-, and reengagement-process. In doing so, the stop-process is very likely the same process involved in the stopping of motor responses and runs in serial with the reengagement process [10]. The advantage of the SCT is that it is more ecologically valid, since situations in everyday life do not only require to stop a single action but also to adaptively change reactions according to environmental requirements. To the best of our knowledge, the present study is the first study which investigated the mechanisms and neural basis of selective changing. Accordingly, it is unclear whether the same selective strategies are used in situations in which the response of one hand is changed while the response with the other hand is continued. However, because of the parallels in global stopping and changing, we assumed that previously available information would trigger the more selective *first discriminate*, *then stop* strategy compared to uninformed situations where presumably the *stop all*, *then discriminate* strategy is used.

## Materials and methods

### Participants

A total of 24 male participants were enrolled in this study. We focused on male participants since gender effects were found in the regions of interest (e.g. caudate nucleus) [11]. Two subjects were excluded because of significant scan artifacts and data acquisition problems. Four participants were identified as outliers on the measures of the SCT. Reasons for excluding those participants are described in more detail in the Methods section. Excluding the data from these six participants, a total of 18 male volunteers aged 19–30 years were left for analysis. Prior to the study the fMRI suitability was clarified in a telephone interview. All participants were students, right handed, had normal, or corrected to normal vision (including normal color vision), and no history of neurological or mental disorders. Moreover, participants with current use of any psychoactive drug were excluded. Before the session, written informed consent was obtained from all participants after complete description of the study. Participants received a monetary reward of €20 in return of the two-hour lasting study. The study protocol was approved by the local ethics committee of the Medical Faculty RWTH Aachen University.

### Stop-change task (SCT)

To test the hypotheses, a bimanual SCT which included cues to trigger the two strategies was used. The task consisted of two different trial types: go- and change-trials. Go-stimuli were two filled green squares which appeared side by side in the middle of the screen. Once the go-signal appeared participants had to press two buttons as fast and simultaneously as possible with both index fingers. In some trials the go-signal was followed by a change-signal after a variable delay (stimulus onset asynchrony, SOA). This change-signal was represented by a red filled square which replaced one of the two green squares of the go-signal. In the case of a change-trial, participants had to inhibit their go-response (e.g. stop pressing the left button with the left index finger) and instead react with the middle finger of the corresponding hand (e.g. pressing the left button with the left middle finger). The reaction with the other hand remained the go-response with the index finger and should still be executed as fast as possible.

**Fig 1:**
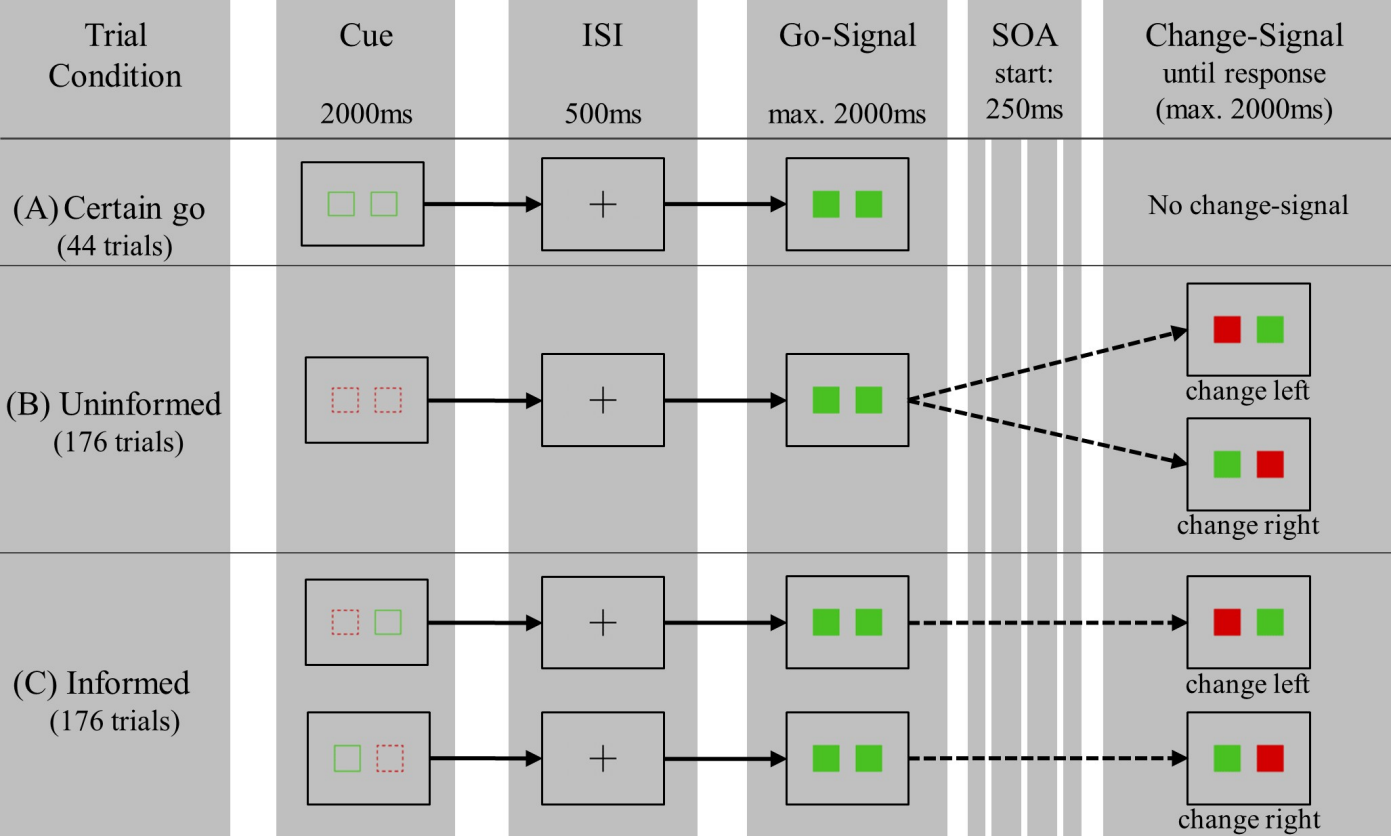
Schematic overview of trial types and conditions in the stop-change task. In the schematic overview each computer screen is represented by a rectangle. The stop-change task consisted of go- and change-trials. In go-trials participants had to respond to the go-signal (two green squares) by pressing simultaneously two corresponding buttons with the index fingers of each hand as fast as possible. In change-trials, after a variable stimulus onset asynchrony (SOA) a change signal (a green and a red squares) was presented after the go signal (two green squares). Depending on the location of the red square participants had to stop pressing the corresponding button with their right or left index finger and should use their middle finger of the right or left hand instead. Go- and change-trials were presented in a mixed design within three foreknowledge conditions. In the certain go condition (A) participants were informed by two green square frames that in the following trial no change signal will appear. In the uninformed condition (B) they were informed by two dashed red square frames that no information was available on which side a change-signal could follow the go-signal. In the informed condition (C) they were informed by a dashed red square frame on which side a change-trial could follow the go-signal. The side of the dashed red square frame corresponds with the hand where the response with index finger has to be stopped and where the middle finger should be used to press the button. ISI = inter-stimulus interval; SOA = stimulus onset asynchrony.

To manipulate foreknowledge three different conditions were available which were presented in a mixed design: certain go (see Fig 1A), uninformed (see Fig1B), and informed (see Fig 1C). The three conditions differed in the previously available information by means of a cue preceding the go-signal in each trial. The cue of the certain go condition consisted of two green squared frames. In this condition participants could be sure that no change-signal would appear in this trial. In the informed condition a red dashed frame and a green frame indicated which hand (the hand corresponding to the red frame) had to change the response in case of a change-trial. In the uninformed condition two red dashed frames indicated that a change-signal could appear. In this condition participants did not know with which hand they might have to execute the alternative response in case of a change-signal.

In the change-trials a staircase-tracking procedure [12] was used for the SOA, targeting an equal distribution of successful and failed change-trials. The delay, starting with 250 ms, increased after a successful change-trial and decreased after a failed change-trial by 50 ms [13]. This procedure made a successful change more likely in the next change-trial after a failed change-trial and vice versa. The SOA was individually adapted for each change-condition and hand.

The stop change paradigm was composed of 396 trials which were split to 44 certain go (11%), 176 informed (44.5%) and 176 uninformed trials (44.5%). In the informed and uninformed condition change-trials made up 25% (i.e. 44 trials), respectively, resulting in an overall probability of 22% change-trials. Trials were presented in a mixed design. The duration of each trial took 5000 ms and was followed by an average jitter of 300 ms. The detailed timing of stimuli is presented in Fig1. In addition to the 396 trials, 40 baseline events were included with the same duration as the task trials. During baseline a blank screen was presented. Overall, the task consisted of 436 trials which resulted in a duration of 41 minutes of measurement.

The selective stop-change task was introduced by a computerized and standardized instruction. Trial-by-trial feedback was given in the training but not during the experiment. The instruction emphasized that both speed and accuracy were important. However, participants were instructed not to wait for change-signals. The stimuli were presented using Presentation software (Version 18, www.neurobs.com) on a Windows system.

### Data analyses

#### Behavioral data

Analysis of behavioral data followed recommendations from literature [14–16]. Prior to data analysis, the RTs of the go- and change-responses that exceeded two standard deviations of the mean of the respective response type were considered as outliers for each participant individually. No more than 11% outliers were identified for any of the response types. For the calculation of mean failed change-RTs only RTs were included in which participants responded with a go-reaction instead of the change-reaction. Trials in which participants did not respond or responded more than once (e.g. responded with index and middle finger of one hand) were not included in this measure.

Participants were excluded from group analysis if the rate of successful change-trials (change accuracy, p[change|signal]) was lower than 30% or larger than 70% and if the proportion of correct go-trials (go accuracy) was less than 90%. Go-trials were classified as error if the time interval between the response executions of both index fingers deviated more than 70 ms or if participants responded with a change- instead of a go-reaction. Moreover, participants were excluded if any variable deviated more than two standard deviations from the groups mean CSRT or go-RT.

The assumptions of the independent horse race model were tested as recommended by Verbruggen and Logan [16]. The change-signal RT (CSRT) is a measure for inhibition time which was calculated using the integration method [15]. The interference of changing (CIE), as a measure of selectivity, was calculated by subtracting the median go-RT of analogous go-trials from the median go-RT of the non-changing hand in a successful change-trial for each condition. The analogous go-trials were determined by rank-ordering the go-RTs and averaging those RTs longer than the nth where n is obtained by multiplying the number of go-RTs in the distribution by the probability of failing to change on change-trials [4]. A positive value of this selectivity measure indicates that go-responses in change-trials were slower as go-responses in go-trials. Preparation costs were calculated by the difference between informed/uninformed go-trials and certain go-trials. Following Chikazoe, Jimura [17] this is a measure for preparatory processes which is associated with inhibitory control.

Additionally, mean go-RTs, mean change-RT (CRT, timespan between change-signal and change-response), mean go RT of the non-cued hand in change-trials (RT of non-changing hand), failed change RTs (RT in change trials in which participants responded with a go response), and mean SOA are reported for each condition. All key measures did not differ between hands and were collapsed across the left and right hands for the informed, uninformed and certain go-condition.

The statistical analyses were computed using SPSS 24.0 for Windows (IBM SPSS Statistics, released 2016). Correlations are Pearson’s correlations. Differences between conditions were examined with paired sample t-tests. In addition, effect sizes and confidence intervals (95% CI) were calculated to estimate the magnitude of the differences between the investigated conditions. As recommended by Dunlap, Cortina [18] the effect size was calculated for independent variables instead of dependent variables as effect sizes for dependent variables often overestimate the actual size of effect.

#### fMRI

The fMRI was performed at the University Hospital of the RWTH Aachen University on a 3 Tesla Siemens Prisma resonance scanner with standard head coil and foam padding to restrict movements. A rear-viewing reflecting mirror was mounted on the MR head coil facing a screen placed at the back end of the scanner. The functional T2* whole brain images were acquired using gradient-echo planar imaging (EPI) parallel to AC/PC-line to detect the changes in blood flow associated with brain activity (BOLD = blood oxygen level-dependent). The following parameter were used for the EPI sequence: echo time (TE) = 30 ms, repetition time (TR) = 2200 ms, flip angle = 90°; image matrix 64 x 64, slice number = 32; slice thickness = 3 mm. Images were acquired in an interleaved order from top to bottom, beginning with even numbers. Stimuli were presented in a rapid event-related design in which a number of 1120 volumetric whole brain images were obtained for each participant. A high-resolution magnetization prepared rapid gradient echo (MPRAGE) structural scan image (TR = 1900 ms, TE = 2.21 ms, flip angle = 9°, image matrix 256 x 256, slice number = 192; slice thickness = 1 mm) was acquired for registration and normalization of the EPI-images.

The brain imaging data were analyzed using SPM12 (Wellcome Department of Imaging Neuroscience, University College London, London). The preprocessing was done in the following order: slice timing correction, realignment, coregistration, normalization, and smoothing.

The first three images of each fMRI run (prescan period) were discarded to eliminate inhomogeneities of the magnetic field at the beginning of the scan. To compensate for differences in time of slice acquisition, slice timing correction to the 16th slice was performed, using SPM12’s Fourier phase shift interpolation. Subsequently, images were motion corrected by realignment process. All T2* images were registered to the mean EPI volume by 2nd degree B-spline interpolation. The structural image was coregistered to the mean EPI image using normalized mutual information. The motion parameters were controlled for each subject. The coregistered images were normalized to the ICBM space template for European brains included in SPM12. As final step of preprocessing the normalized images were smoothed with 8 mm full-width half maximum (FWHM) Gaussian distribution.

A first level event-related analysis was done for each subject using a general linear model (GLM). The cue, go-signal, and change-signal were used as explanatory variables. We separately modeled these events with regard to trial type (go, change), condition (certain, informed, uninformed), and correct or false responses. Thus we modeled 12 conditions for go trials (2 [cue/go-signal] x 3 [certain/informed/uninformed] x 2 [correct/false]) and 12 conditions for change trials (3 [cue/go-signal/change-signal] x 2 [informed/uninformed] x 2 [correct/false]). In addition, the null-event was modeled as a baseline factor. To account for residual head motion effects, the motion parameters from the realignment procedure were included into the statistical model. Each GLM was estimated separately for each participant. Our primary interest was on correct change and preparatory processes in informed and uninformed trials. We contrasted activity during change-signal and cue for informed and uninformed trials with activity during baseline to correct for task-irrelevant activity. For change, only images from correct change responses were used. Please note, we investigated both the contrast versus baseline and the contrast versus the certain go-signal. As results were merely the same, we decided to report the first option since the second one follow the assumption that activity during go and stop/change processes are independent from each other. In this complex task, in which participants should respond with both index fingers on go-signals and change their response of only one hand to a response with the middle finger, the contrast versus baseline is probably the more conservative with regard to the independence assumption. To obtain group statistics each participant’s contrast image was entered into a second-level random-effects analysis using a full factorial design (Cue[informed/uninformed] x Event [cue/change]). For whole brain analyses significance was accepted for clusters exceeding a statistical threshold of p < 0.05 family-wise error (FWE) corrected.

Furthermore, a region of interest (ROI) approach was used to examine the areas for which a priori hypotheses were formulated. The binary mask for the ROI analysis including the caudate nucleus and STN were created using the Wake Forest University Pick Atlas (version 3.0.4) [19] and the integrated AAL [20] and IBASPM 71 (for STN mask; http://www.thomaskoenig.ch/Lester/ibaspm.htm) atlases. For the ROI analyses, significance was accepted for a statistical threshold of p < 0.05 FWE corrected on small volume. Beta values for the ROIs were extracted using MarsBaR [21] and Pearson’s correlations were calculated with behavioral measures.

All activation loci reported in this work as MNI coordinates were verified using the Anatomy software (Version 2.1) [22]. The single subject anatomical T1 image from SPM12 was used for visualization of statistical parametric maps. Figures were created using the Multi-image Analysis GUI (Version 3.8; Mango, Research Imaging Institute, UTHSCSA).

## Results

### Behavioral results

#### Accuracy, staircase procedure, and independence assumption

Mean RTs in the SCT are presented in Table 1. Participants achieved a mean successful change rate of 49% in the informed condition and 48% in the uninformed condition with a mean SOA of 242 ms (*SD* = 86) for the informed condition and 217 ms (*SD* = 100) for the uninformed condition. A successful change rate of ~50% is an evidence for a successful application of the staircase procedure. The independence of go and change processes was confirmed by inspecting the go-RTs and failed change-RT (non-changing hand) for each participant. For each participant the average go-RT in failed change-trials was shorter compared to the corresponding go-RT.

**Table 1:** Reaction times (mean, standard deviation) for go and change trials separated for trials with (informed) and without foreknowledge (uninformed).

Behavioral measure	(Mean ± SD)						
Certain-go RT			337	±	72		
% of certain-go errors			9.6	±	4.4		
	Informed				Uninformed		
Go RT	565	±	109		558	±	116
% of go errors	5.2	±	4.1		2.4	±	3.1
Change RT	432	±	92		527	±	121
CSRT	312	±	30		334	±	36
Failed change RT (non-changing hand)	498	±	82		473	±	91
Failed change RT (go hand)	498	±	81		468	±	91
CIE	31	±	72		102	±	99
Preparation Cost	228	±	112		221	±	114
% of correct change	48.6	±	7.9		47.6	±	8.3
SOA	242	±	86		217	±	100

Failed change RT is the RT in change trials in which participants responded with a go response. CIE = change interference effect; CSRT = change signal reaction time; RT = reaction time; SOA = stimulus onset asynchrony.

#### Informed versus uninformed selective changing

The paired sample *t*-test revealed that participants benefitted from the information in the informed condition which became visible in a significantly shorter CRT (*t*(17) = -5.36, *p* < .001, *d* = -0.89, *CI*_*d*_ [-1.55, -0.19]), CSRT (*t*(17) = -4.13, *p* < .001, *d* = -0.67, *CI*_*d*_ [-1.33, 0.02]) and CIE (*t*_(17)_ = -3.85, *p* < .001, *d* = -0.82, *CI*_*d*_ [-1.48, -0.12]).

#### Informed, uninformed and certain go

Participants responded significantly faster in the certain go trials compared to informed (*t*(17) = -8.61, *p* < .001, *d* = -2.47, *CI*_*d*_ [-3.27, -1.55]) and uninformed (*t*(17) = -8.24, *p* < .001, *d* = -2.29, *CI*_*d*_ [-3.07, -1.41]) go trials. Go RTs during the informed and uniformed condition did not significantly differ (*t*(17) = 1.29, *p* = .216, *d* = 0.06, *CI*_*d*_ [-0.59, 0.71]).

The preparation costs, describing the difference between certain and informed/uninformed go RTs, did not significantly differ between conditions (*t*(17) = 1.19, *p* = .249, *d* = 0.06, *CI*_*d*_ [-0.59, 0.72]).

#### Selectivity is associated with preparation costs

An additional analysis yielded a significant negative correlation between the CIE and preparation cost but only in the informed condition (*r* = -0.51, *p* < .05) and not in the uninformed condition (*r* = -.12, *p* < .64). A smaller CIE indicates higher selectivity. Accordingly, high selectivity accompanies with high preparation cost for inhibition in the informed condition.

### Neuroimaging results

#### Changing

**Fig 2:**
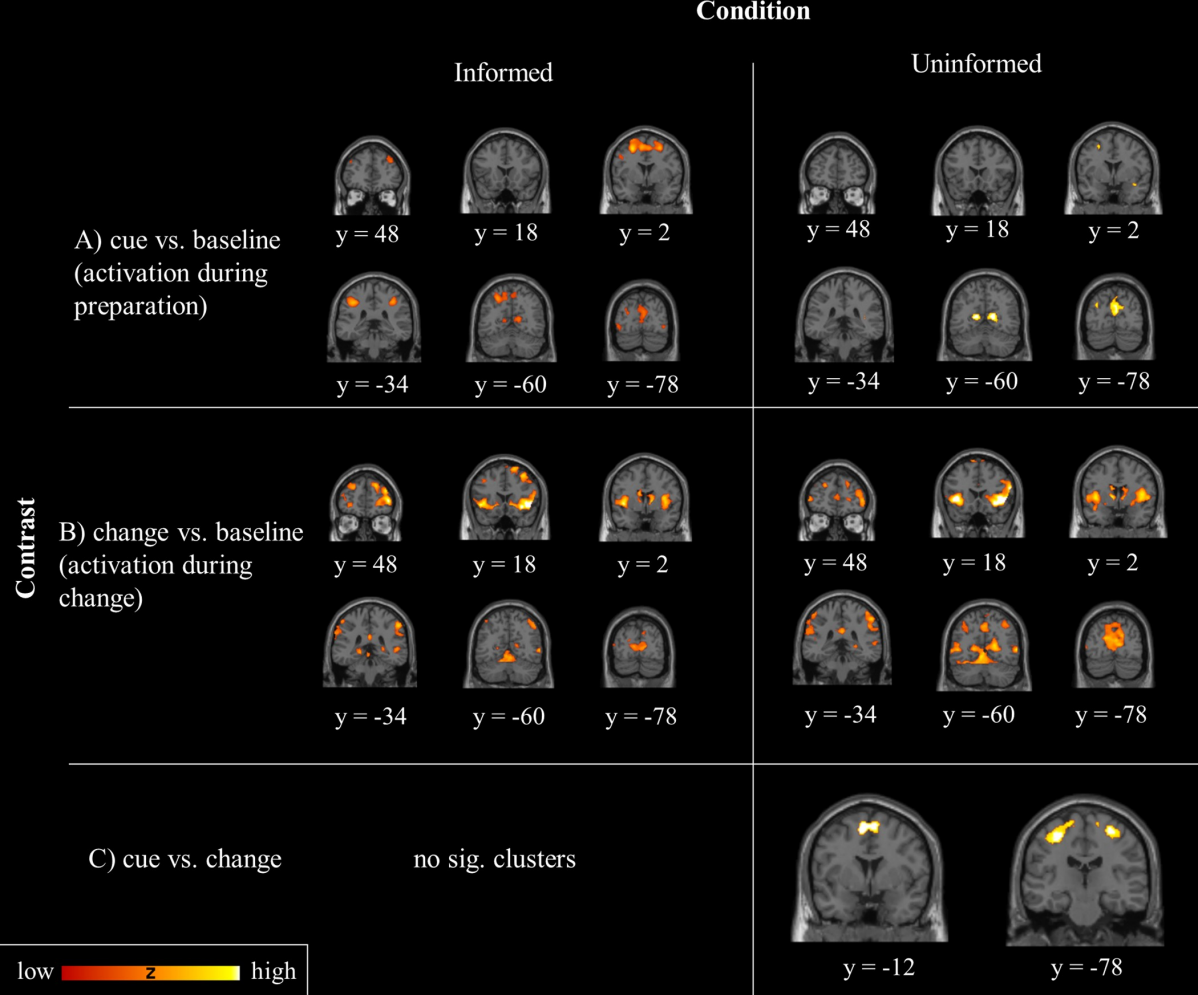
Activation during cue and change-signal presentation in the informed and uninformed condition. Rows present activation for the contrasts: A) cue vs. baseline, B) change vs. baseline, C) cue vs. change for the informed (left column) and uninformed (right column) condition. More reddish blobs represent lower Z values, while more yellowish blobs represent higher Z values. Slices are presented from rostral to caudal with y-coordinates assigned to MNI space. All activations were uncorrected on voxel level (*p* <0.0001, spatial extent threshold of 20 adjacent voxels).

We first investigated neural activity during the correct change process. In both informed and uninformed changing we found significant BOLD signals in the right IFG, right Insula, and bilateral calcarine gyrus and anterior and middle cingulate cortex. Additionally, in the informed condition significant activations were found in the right inferior parietal lobule and the medial, middle and superior frontal gyri. In the uninformed condition, we found significant activations in the superior and middle temporal gyrus (see Fig 2 and Table 2). However, when contrasting the informed and uninformed conditions during changing, we did not find any significant clusters. ROI analyses revealed that in both informed and uninformed changing the left and right caudate nucleus (part of indirect pathway) showed significant BOLD responses (informed: x = -12, y = -2, z = 20, *t* = 4.78 and x = 14, y = 16, z = -4, *t* = 5.11; uninformed: x = -10, y = 4, z = 4, *t* = 5.65 and x = 12, y = 6, z = 4, *t* = 5.09). We did not find any significant activations in the STN (part of hyperdirect pathway). The beta values for the left caudate nucleus during informed correct changing significantly correlated with CSRT (*r* = .496, *p* = .036). There were no significant correlations between beta values of left or right caudate nucleus and CIE or CRT for informed changing and between beta values and CSRT, CIE, or CRT for uninformed changing.

**Table 2:** Brain regions showing signal increases during the change process.

Contrast	Anatomical label	Hem	Cluster size (mm^2^)	MNI coordinates (mm)			t-value
				x	y	z	
Informed changing	Insula lobe / IFG (p. Orbitalis) / Inferior parietal lobule	R	14124	46	18	-4	7.57
				34	22	-8	7.02
				52	-44	52	6.90
	Cerebellum	R/L	1558	12	-50	-16	6.67
				-12	-50	-16	6.56
				0	-44	-16	5.41
	Anterior cingulate cortex	R	956	4	34	28	5.83
				6	42	4	4.09
	Superior medial gyrus / Superior frontal gyrus	R	708	16	24	64	5.58
				8	42	52	4.85
				16	22	52	4.02
	Calcarine Gyrus / Lingual Gyrus	R/L	1585	8	-78	6	5.18
				-8	-78	4	4.84
				10	-70	14	4.57
	Middle cingulate cortex	R/L	436	0	-34	26	4.61
				-2	-28	20	4.44
				2	-14	32	4.10
	Middle frontal gyrus	L	280	-20	48	32	4.55
				-36	44	34	4.45
	Middle frontal gyrus	L	268	-44	28	36	4.51
				-38	24	42	4.23
				-34	12	44	3.91
Uninformed changing	Insula lobe / IFG (p. Opercularis)	R/L	13661	42	16	0	7.86
				52	20	-2	7.72
				-38	20	0	7.55
	Calcarine gyrus / Cerebelum	L	8145	16	-66	18	6.39
				-14	-50	-18	6.35
				-10	-70	12	5.83
	Middle temporal gyrus / Superior temporal gyrus	L	468	60	-60	6	5.50
				60	-50	2	5.42
				64	-38	8	3.82
	Middle cingulate cortex	L	312	2	-32	30	5.30
				2	-30	40	3.55
	Anterior cingulate cortex	R	896	0	30	24	4.86
				0	40	22	4.58
				2	42	8	4.26

All contrasts are against baseline. Significance was accepted for clusters exceeding a statistical threshold of p< .05 family-wise error (FWE) corrected and k > 20.

#### Preparation of changing

As described, we found a negative correlation between selectivity (CIE) and preparation cost indicating that participants who delayed their response to a go-signal in expectation of a possible change-signal showed a smaller CIE in change trials. This relationship could be an indicator for preparatory processes. Thus, we focused in the following exploratory analyses on the cue (preparation phase) and investigated activation during informed and uniformed trials at this point of time. We found occipital activations in both conditions. However, we only found prefrontal activations (superior frontal gyrus) in the informed preparation phase, while during the uninformed preparation phase solely the primary sensory cortex was activated (see Fig 2 and Table 3). When contrasting the informed and uninformed conditions, the analysis did not reveal any significant clusters. In the ROI analysis we did not find any significant activations in caudate nucleus or STN. When contrasting the activation between preparation and change processing to identify regions which are active in addition to the regions involved in the change processing, we found no significant activations for the informed condition. However, in the uninformed condition, we found additional activation in comparison to the activations in uninformed changing in bilateral M1 and posterior medial frontal gyrus.

**Table 3:** Brain regions showing signal increases during the preparation process.

Contrast	Anatomical label	Hem	Cluster size (mm^2^)	MNI coordinates (mm)			t-value
				x	y	z	
Informed preparation	Superior frontal gyrus	R/L	3558	24	-6	54	9.23
				-24	-6	54	8.21
				-24	-4	70	5.62
	Postcentral gyrus / Superior parietal lobule	L	1745	-40	-32	42	5.56
				-26	-58	50	4.87
				-38	-38	52	4.79
	Postcentral gyrus / Superior parietal lobule	R/L	599	36	-32	44	5.12
				28	-52	64	4.01
				28	-48	46	3.99
	Cuneus / Calcarine gyrus	R/L	1312	4	-86	24	4.65
				2	-88	12	4.44
				-10	-68	12	4.40
	Calcarine gyrus / Inferior occipital gyrus	L	470	-14	-94	-10	4.46
				-18	-100	-6	4.36
				-24	-90	-10	4.34
	Middle occipital gyrus / Inferior occipital gyrus	R	457	28	-98	8	4.25
				36	-94	0	4.11
				44	-84	-2	4.09
Uninformed preparation	Lingual gyrus / Precuneus / Cuneus	R/L	2410	12	-46	4	4.86
				-10	-48	8	4.85
				4	-88	24	4.84
	Calcarine gyrus / Lingual gyrus / Inferior occipital gyrus	R	498	24	-92	2	4.56
				42	-86	-2	4.14
				36	-94	0	4.00
	Inferior occipital gyrus	L	238	-24	-88	-10	4.35
				-18	-96	-8	4.14
				-4	-86	-8	3.54
Uninformed: Preparation vs. Changing	Posterior-medial frontal / Precentral gyrus	L	1627	-6	-4	60	5.68
				-34	-16	54	5.26
				-28	-26	54	4.88
	Precentral gyrus	R	373	32	-18	60	4.40
				50	-10	58	4.31
				36	-12	56	3.82

Contrast for informed and uninformed preparation are against baseline. Significance was accepted for clusters exceeding a statistical threshold of p< .05 family-wise error (FWE) corrected and k > 20.

## Discussion

The aim of the present study was to investigate the behavioral and neural effects of different kinds of foreknowledge on selective stopping and changing. We assumed that the fast but global *stop-all*, *then discriminate* strategy would be used in uninformed situations and would be processed via the hyperdirect pathway including the STN. The more selective *first discriminate*, *then stop* strategy was suggested to be processed via the indirect pathway including the caudate nucleus, especially, when foreknowledge about the possible later changing was available.

The comparison between informed and uninformed changing on the behavioral level revealed that informed changing was advantageous in selectivity (CIE), inhibition speed (CSRT), and in change RT (CRT). Accordingly and in contrast to our assumption, the uninformed condition did not lead to faster inhibition times (CSRT), even though the change process was more global (less selective, measured by CIE) in the uninformed condition. However, it has to be mentioned that besides the significant paired t-test, the confidence intervals of Cohen’s *d* for the comparisons between informed and uninformed CSRTs included the zero indicating that the result might not be statistically significant. Additionally, we found that selectivity was correlated with preparation cost, but only in the informed condition. Participants with higher preparation costs were able to change their response more selectively.

The imaging data showed that informed and uninformed changing rarely differed in neural processing. In both conditions activations were found in the right IFG, insula, and cingulate gyrus. The ROI analysis did not reveal any activation in the STN in neither condition. However, we found significant BOLD responses during selective changing in the caudate nucleus. This leads to the assumption that in both conditions the selective indirect pathway was used. Activation in caudate nucleus during informed changing, but not uninformed changing, was associated with inhibition time (CSRT). Based on the behavioral results we assume that the differences in selectivity and inhibition speed are a result of different preparation processes. Therefore, we examined the differences in neural activity during the preparation phase of informed and uninformed changing. However, no significant difference was found in direct comparison between conditions. Further, we investigated the activity during informed and uninformed preparation phase when contrasted with the activity during the change process. Here we did not find any significant clusters for informed changing, while uninformed changing revealed significant BOLD responses in the M1 and posterior medial frontal (including preSMA/SMA). Those are action execution areas. Apparently in the uninformed condition, an appropriate motor set was created.

The brain regions found active during the change processing have been consistently found in other studies with inhibition-tasks [1, 23–28]. Several studies (e.g. [5, 6]) could show that information in a stop-signal task promotes a more selective inhibition mechanism. Aron and Verbruggen [4], therefore, suggested that this informed selective inhibition would be processed via the selective indirect pathway and uninformed selective inhibition via the fast and global hyperdirect pathway. Our behavioral data showed higher selectivity for the informed condition. However, on a neural level we could not distinguish between different basal ganglia pathways used for its processing. The indirect pathway (caudate nucleus) which is assumed to process the *first discriminate*, *then stop* strategy was used irrespective of the level of information. In contrast to the assumption by Aron and Verbruggen [4], we did not find faster inhibition times and no STN activation during uninformed changing. Smittenaar, Guitart-Masip [29] replicated the study by Aron and Verbruggen [4] with small adaptions in an fMRI study. Similar to our results, they found a beneficial effect in informed trials on both inhibition time and selectivity. Moreover and contrary to the assumption by Aron and Verbruggen [4], they found that speeded stopping rather than selectivity was associated with activity in dlPFC and striatum (including caudate nucleus). The parametrical analysis of beta values in our ROI analysis confirms the result by Smittenaar, Guitart-Masip [29]. We found a significant correlation between caudate nucleus activation during informed changing and inhibition time (CSRT). These results indicate that not only the STN in the global hyperdirect pathway might promote fast inhibition processes, but also the caudate nucleus when preparation due to foreknowledge is possible.

The fast hyperdirect pathway has been consistently found in simple SSTs in which participants had to stop one action. Compared to these simple SSTs, the bimanual SCT used here is much more complex. In our task participants had to stop the ongoing response and also to initiate and execute another motor response. Furthermore, they had to selectively change only the response of one hand and had to continue the go response with the other hand. In addition, they had to consider the presented cues which informed them that an appearance of a stop-signal is possible but not certain.

It is possible that the STN was not found to be significantly active in this task due to the poor spatial resolution of the 3 Tesla scanner and due to the small sample size. However, in earlier studies in which simple stop-signal tasks or unimanual stop-change tasks were used, a STN activation was consistently found with similar methods and samples sizes [1, 8, 30]. Nevertheless, our findings seem to indicate that the indirect pathway was used in the SCT. We assume that the tasks complexity and possibility to prepare for the stop process in both conditions had an influence on the involved neural pathways (especially the indirect pathway). Both factors are likely to have an influence on which strategy was used to accomplish the SCT.

The measure for selectivity (CIE) in the informed condition was correlated with preparation costs. This correlation was not present in the uninformed condition. Moreover, conditions only differed in selectivity and not in preparation costs. We assume that differences in neural processing during the preparation phase may be responsible for the more selective inhibition in the informed condition. It is conceivable that the preparation in the informed condition was more specific. In a previous study it has been shown that efficient preparation is associated with advanced activations of the inhibition network [17]. We did not find any significant neural differences between informed and uninformed changing during the preparation phase. When investigating whether participants activated the inhibition network in advance as preparatory process, we also did not find any significant differences between activations during preparation and changing in the informed condition. This indicates that participants efficiently activated the regions for changing in advance. This is in line with studies [17, 29, 31] which accounted for the advanced activation of the inhibition network for preparatory purposes. However, in the uninformed preparation phase participants showed additional activity to the change activity in medial frontal gyrus (preSMA) and M1. The missing information in the uninformed condition may have led to a more inefficient preparation to change the response. These results can be interpreted in a way that in the informed condition participants were one step ahead during the informed preparation phase: the activations during informed preparation overlap with activations of change-processing indicating that the change-processes and the go-process of the non-changing hand were initiated in advance which presumably led to a fast and more selective inhibition process.

Moreover, our findings are in line with the dual mechanism framework by Braver [32] who argued that in reactive inhibition additional processes are needed for successful action control. In a recent patient study by Roberts and Husain [33] it was shown that the preSMA is especially important for rapidly updating and implementing response plans and, therefore, for response changing. The authors compared behavioral measures of stop-signal task, stop change task and Eriksen flanker task between a patient with a lesion of the preSMA and healthy controls. While the patient did not show any impairment in response stopping (SSRT) the time needed to inhibit a response in the stop change task (CSRT) was significantly longer compared to control participants. Presumably the additional preSMA activation in uninformed control processes found in the present study was used to rapidly update the response plan as soon as the change-signal appeared on the screen. Moreover, it can be assumed that the preparation in the uninformed condition was set up to prepare for fast target detection so that the change process can be reactively performed as soon as it is clear which response the participant had to perform. These additional processes were unnecessary in the informed condition as the additional information enabled the preparation for the concrete action. In the course of discussion about the interaction between IFG and SMC and their concrete role in inhibition processing, the results presented here indicate that the preSMA is important for setting up the inhibition network [34–36] and that the IFG implements the action control [27]. To prove this hypothesis further, more time sensitive analysis methods would be needed.

Besides the effect of foreknowledge on preparatory processes, it is also conceivable that the benefit of foreknowledge resulted from motivational changes in the participants. This aspect was considered by Leotti and Wager [37] who investigated whether the SSRT is truly independent of external influences such as participants’ expectations and motivations. In four experiments the authors were able to show that inhibitory performance can be influenced by motivational factors (i.e. monetary incentives or punishment for fast go-trials and correct inhibition) and explicit strategic control. The motivational bias is reflected in the individual differences in the trade-off between fast responding and accurate inhibition. A study by Padmala, Pessoa [38] investigated the same topic with a stop-signal task with and without reward for correct go-responses. In this study rewarding correct go-responses led to longer SSRT compared to the condition without reward, indicating motivation impaired inhibitory processing rather than facilitated the execution of prepotent responses. On the neuronal level interactions between rewarding-go and stop-task inhibition was found for cortical and subcortical regions such as the IFG and putamen. It is, therefore, conceivable that the information given in our informed condition also had a challenging impact to respond as fast as possible.

Finally, it cannot be said if the participants’ motivation or strategies were influenced by foreknowledge. However, the broad overlap between preparation and change activations for the informed condition and the fact that the change-processes did not differ between conditions let suggest that the informed condition above all benefitted from the pre-activation of relevant brain regions.

In this study, as well as in the majority of stop-signal task studies, the time needed to inhibit a response (SSRT, CSRT) was of particular interest. The problem of inhibition research is that the behavior in focus cannot be directly observed. By estimating the SSRT or CSRT with the mathematical horse race model only an approximation of the stopping latency can be made [39]. Concomitantly, this emphasizes the advantage of the proactive selective stop change task over the stop-signal task: the stop change task enables the direct measure of the CRT and is, therefore, not completely dependent on the horse-race model.

A further issue is the poor spatial resolution of 3 Tesla fMRI. This becomes especially problematic with the investigation of very small structures such as the STN. Moreover, the interpretation for ROI analyses is dependent on the atlas used for the analysis [40], we repeated the ROI analyses with a box (10x10x10 mm) at the coordinates identified by Forstmann, Keuken [41] which lead to the same null effect found in the previous analysis. Accordingly, the null effect of STN activation can be understood as an indicator for no or less involvement of the STN in selective changing. To finally exclude an involvement of the STN and hyperdirect pathway in this complex SCT, the study should be replicated in with ultra-high field MRI which enables higher spatial resolution [42].

### Conclusion

Findings of this study indicate that strategies used for selective inhibition seems to be dependent on the complexity of the task and therefore on the activation of different motor programs. Our results indicate, that the neural network involved in those complex operations include the indirect pathway which is assumed to be the neural correlate of the *first discriminate*, *then stop* strategy. The global hyperdirect pathway and *stop all*, *then discriminate* strategy might be used in more easy tasks or tasks in which speed is more important than selectivity. Preparatory processes due to foreknowledge have led to faster and more selective change responses.

## References

[1] Aron AR. From reactive to proactive and selective control: Developing a richer model for stopping inappropriate responses. Biol Psychiatry. 2011;69(12):e55–68. 10.1016/j.biopsych.2010.07.024 20932513 PMC3039712

[2] Alexander GE, Crutcher MD, DeLong MR. Basal ganglia-thalamocortical circuits: Parallel substrates for motor, oculomotor, "prefrontal" and "limbic" functions. Prog Brain Res. 1990;85:119–46. . 2094891

[3] Nambu A, Tokuno H, Takada M. Functional significance of the cortico-subthalamo-pallidal 'hyperdirect' pathway. Neurosci Res. 2002;43(2):111–7. . 12067746 10.1016/s0168-0102(02)00027-5

[4] Aron AR, Verbruggen F. Stop the presses: Dissociating a selective from a global mechanism for stopping. Psychol Sci. 2008;19(11):1146–53. 10.1111/j.1467-9280.2008.02216.x . 19076487

[5] Claffey MP, Sheldon S, Stinear CM, Verbruggen F, Aron AR. Having a goal to stop action is associated with advance control of specific motor representations. Neuropsychologia. 2010;48(2):541–8. 10.1016/j.neuropsychologia.2009.10.015 WOS:000274371700021. 19879283 PMC2813913

[6] Lavallee CF, Meemken MT, Herrmann CS, Huster RJ. When holding your horses meets the deer in the headlights: time-frequency characteristics of global and selective stopping under conditions of proactive and reactive control. Front Hum Neurosci. 2014;8:994 10.3389/fnhum.2014.00994 PMC4262052. 25540615 PMC4262052

[7] Logan GD, Cowan WB, Davis KA. On the ability to inhibit simple and choice reaction time responses: A model and a method. J Exp Psychol Hum Percept Perform. 1984;10(2):276–91. 10.1037/0096-1523.10.2.276 . 6232345

[8] Boecker M, Drueke B, Vorhold V, Knops A, Philippen B, Gauggel S. When response inhibition is followed by response reengagement: An event-related fMRI study. Hum Brain Mapp. 2011;32(1):94–106. 10.1002/hbm.21001 . 20336654 PMC6870390

[9] Boecker M, Gauggel S, Drueke B. Stop or stop-change—Does it make any difference for the inhibition process? Int J Psychophysiol. 2013;87(3):234–43. 10.1016/j.ijpsycho.2012.09.009 . 23026439

[10] Verbruggen F, Schneider DW, Logan GD. How to stop and change a response: The role of goal activation in multitasking. J Exp Psychol Hum Percept Perform. 2008;34(5):1212–28. 10.1037/0096-1523.34.5.1212 . 18823206

[11] Li CSR, Huang C, Constable RT, Sinha R. Gender differences in the neural correlates of response inhibition during a stop signal task. Neuroimage. 2006;32(4):1918–29. 10.1016/j.neuroimage.2006.05.017 WOS:000240969200034. 16806976

[12] Kaernbach C. Simple adaptive testing with the weighted up-down method. Percept Psychophys. 1991;49(3):227–9. . 2011460 10.3758/bf03214307

[13] Verbruggen F, Logan GD, Stevens MA. STOP-IT: Windows executable software for the stop-signal paradigm. Behav Res Methods. 2008;40(2):479–83. 10.3758/brm.40.2.479 . 18522058

[14] Logan GD. On the ability to inhibit thought and action: A users' guide to the stop signal paradigm. 1994.

[15] Verbruggen F, Chambers CD, Logan GD. Fictitious inhibitory differences: How skewness and slowing distort the estimation of stopping latencies. Psychol Sci. 2013;24(3):352–62. Epub 2013/02/13. 10.1177/0956797612457390 23399493 PMC3724271

[16] Verbruggen F, Logan GD. Evidence for capacity sharing when stopping. Cognition. 2015;142:81–95. 10.1016/j.cognition.2015.05.014 . 26036922 PMC4787292

[17] Chikazoe J, Jimura K, Hirose S, Yamashita K, Miyashita Y, Konishi S. Preparation to inhibit a response complements response inhibition during performance of a stop-signal task. J Neurosci. 2009;29(50):15870–7. 10.1523/JNEUROSCI.3645-09.2009 . 20016103 PMC6666181

[18] Dunlap WP, Cortina JM, Vaslow JB, Burke MJ. Meta-analysis of experiments with matched groups or repeated measures designs. Psychol Methods. 1996;1(2):170–7. 10.1037/1082-989X.1.2.170

[19] Maldjian JA, Laurienti PJ, Kraft RA, Burdette JH. An automated method for neuroanatomic and cytoarchitectonic atlas-based interrogation of fMRI data sets. Neuroimage. 2003;19(3):1233–9. 10.1016/S1053-8119(03)00169-1 12880848

[20] Tzourio-Mazoyer N, Landeau B, Papathanassiou D, Crivello F, Etard O, Delcroix N, et al Automated anatomical labeling of activations in SPM using a macroscopic anatomical parcellation of the MNI MRI single-subject brain. Neuroimage. 2002;15(1):273–89. Epub 2002/01/05. 10.1006/nimg.2001.0978 . 11771995

[21] Brett M, Anton JL, Valabregue R, Poline JB, editors. Region of interest analysis using an SPM toolbox [abstract]. 8th International Conference on Functional Mapping of the Human Brain; 2002; Sendai, Japan: NeuroImage.

[22] Eickhoff SB, Paus T, Caspers S, Grosbras M-H, Evans AC, Zilles K, et al Assignment of functional activations to probabilistic cytoarchitectonic areas revisited. Neuroimage. 2007;36(3):511–21. 10.1016/j.neuroimage.2007.03.060 17499520

[23] Bari A, Robbins TW. Inhibition and impulsivity: Behavioral and neural basis of response control. Prog Neurobiol. 2013;108:44–79. 10.1016/j.pneurobio.2013.06.005 . 23856628

[24] Lee HW, Lu M-S, Chen C-Y, Muggleton NG, Hsu T-Y, Juan C-H. Roles of the pre-SMA and rIFG in conditional stopping revealed by transcranial magnetic stimulation. Behav Brain Res. 2016;296:459–67. 10.1016/j.bbr.2015.08.024 26304720

[25] Chevrier A, Cheyne D, Graham S, Schachar R. Dissociating Two Stages of Preparation in the Stop Signal Task Using fMRI. PLoS One. 2015;10(6):e0130992 10.1371/journal.pone.0130992 26110429 PMC4481508

[26] Chevrier AD, Noseworthy MD, Schachar R. Dissociation of response inhibition and performance monitoring in the stop signal task using event-related fMRI. Hum Brain Mapp. 2007;28(12):1347–58. Epub 2007/02/03. 10.1002/hbm.20355 . 17274022 PMC6871417

[27] Jahfari S, Stinear CM, Claffey M, Verbruggen F, Aron AR. Responding with restraint: What are the neurocognitive mechanisms? J Cogn Neurosci. 2010;22(7):1479–92. 10.1162/jocn.2009.21307 19583473 PMC2952035

[28] Li CS, Yan P, Sinha R, Lee TW. Subcortical processes of motor response inhibition during a stop signal task. Neuroimage. 2008;41(4):1352–63. Epub 2008/05/20. 10.1016/j.neuroimage.2008.04.023 ; PubMed Central PMCID: PMCPmc2474693. 18485743 PMC2474693

[29] Smittenaar P, Guitart-Masip M, Lutti A, Dolan RJ. Preparing for selective inhibition within frontostriatal loops. J Neurosci. 2013;33(46):18087–97. 10.1523/JNEUROSCI.2167-13.2013 24227719 PMC3828462

[30] Aron AR, Poldrack RA. Cortical and subcortical contributions to Stop signal response inhibition: Role of the subthalamic nucleus. J Neurosci. 2006;26:2424–33. 10.1523/JNEUROSCI.4682-05.2006 16510720 PMC6793670

[31] Majid DS, Cai W, Corey-Bloom J, Aron AR. Proactive selective response suppression is implemented via the basal ganglia. J Neurosci. 2013;33(33):13259–69. 10.1523/JNEUROSCI.5651-12.2013 23946385 PMC3742918

[32] Braver TS. The variable nature of cognitive control: A dual mechanisms framework. Trends Cogn Sci. 2012;16(2):106–13. 10.1016/j.tics.2011.12.010 22245618 PMC3289517

[33] Roberts RE, Husain M. A dissociation between stopping and switching actions following a lesion of the pre-supplementary motor area. Cortex. 2015;63:184–95. 10.1016/j.cortex.2014.08.004 25282056 PMC4317195

[34] Neubert FX, Mars RB, Rushworth MF. Is there an inferior frontal cortical network for cognitive control and inhibition. Principles of frontal lobe function. 22013. p. 332–52.

[35] Rushworth MFS, Walton ME, Kennerley SW, Bannerman DM. Action sets and decisions in the medial frontal cortex. Trends Cogn Sci. 2004;8(9):410–7. 10.1016/j.tics.2004.07.009 15350242

[36] Swann NC, Cai W, Conner CR, Pieters TA, Claffey MP, George JS, et al Roles for the pre-supplementary motor area and the right inferior frontal gyrus in stopping action: Electrophysiological responses and functional and structural connectivity. Neuroimage. 2012;59(3):2860–70. 10.1016/j.neuroimage.2011.09.049 21979383 PMC3322194

[37] Leotti LA, Wager TD. Motivational influences on response inhibition measures. Journal of experimental psychology Human perception and performance. 2010;36(2):430–47. 10.1037/a0016802 PMC3983778. 20364928 PMC3983778

[38] Padmala S, Pessoa L. Interactions between cognition and motivation during response inhibition. Neuropsychologia. 2010;48(2):558–65. 10.1016/j.neuropsychologia.2009.10.017 19879281 PMC2813998

[39] Boehler CN, Appelbaum LG, Krebs RM, Hopf JM, Woldorff MG. The influence of different Stop-signal response time estimation procedures on behavior-behavior and brain-behavior correlations. Behav Brain Res. 2012;229(1):123–30. Epub 2012/01/17. 10.1016/j.bbr.2012.01.003 ; PubMed Central PMCID: PMCPmc3306010. 22245527 PMC3306010

[40] de Haan B, Karnath HO. 'Whose atlas I use, his song I sing?'—The impact of anatomical atlases on fiber tract contributions to cognitive deficits after stroke. Neuroimage. 2017;163:301–9. Epub 2017/09/30. 10.1016/j.neuroimage.2017.09.051 . 28958880

[41] Forstmann BU, Keuken MC, Jahfari S, Bazin P-L, Neumann J, Schäfer A, et al Cortico-subthalamic white matter tract strength predicts interindividual efficacy in stopping a motor response. Neuroimage. 2012;60(1):370–5. 10.1016/j.neuroimage.2011.12.044 22227131

[42] Forstmann BU, de Hollander G, van Maanen L, Alkemade A, Keuken MC. Towards a mechanistic understanding of the human subcortex. Nat Rev Neurosci. 2017;18(1):57–65. 10.1038/nrn.2016.163 27974841

